# New developments in the pathology of malignant lymphoma: a review of the literature published from May to August 2017

**DOI:** 10.1007/s12308-017-0303-1

**Published:** 2017-09-30

**Authors:** J. H. van Krieken

**Affiliations:** Department of Pathology, Radboud University Medical Centre, P.O. Box 9101, 6500, HB Nijmegen, The Netherlands

## Introduction

This quarterly review deals with quite a substantial series of articles that present data which are relevant for our view on lymphoma development and on application of the information in our routine work. A remarkable series of articles deal with EBV or MYC.

## Biology of lymphoma

The idea of pre-lymphoma stem cells has arisen, because translocations involving antigen receptor genes are due to mistakes in events that occur in early B and T lymphocytes in the bone marrow, while the resulting lymphomas consist of mature cells with additional genetic alterations. Qing et al. [1] develop a model that gives further proof for this hypothesis. They use mismatch repair deficient mice with a MSH2 −/− phenotype. In humans, this phenotype results in aggressive gliomas and a variety of lymphomas and leukemias. They show that after bone marrow transplantation with BM cells from MSH2−/− mice there is full reconstitution of multiple hematopoietic lineages in lethally irradiated wild-type recipients. All recipients developed thymic lymphomas; however, lymphomas did not occur in thymectomized recipients. These results indicate that early T cells with a MSH −/− defect carry a lymphomagenic potential that is dependent on the thymic microenvironment. This model may apply for other early defects and lymphoma types too.

### Hodgkin lymphoma

Classical Hodgkin lymphoma (cHL) has been studied extensively, but still new mechanisms are linked to it. Li et al. [2] investigated the role of monoamine oxidase A (MAOA), a mitochondrial enzyme that catalyzes oxidative deamination of neurotransmitters and dietary amines and produces H_2_O_2_. MAOA was expressed by Hodgkin Reed-Sternberg (HRS) cells in the majority of cHLs (181/241, 75%), with 35% showing strong expression. Weak MAOA was also noted in a minority of primary mediastinal diffuse large B cell lymphomas (mDLBCL, 8/47, 17%) and in a mediastinal gray-zone lymphoma. No MAOA was found in non-neoplastic lymphoid tissues, nodular lymphocyte-predominant Hodgkin lymphoma (NLPHL, 0/8), or any other non-Hodgkin lymphomas studied (NHL, 0/123). MAOA was more common in Epstein-Barr virus (EBV)-negative compared to EBV-positive cHL. Most cHL-derived cell lines displayed MAOA activity as well, whereas NHL-derived cell lines did not. The MAOA inhibitor clorgyline reduced the growth of L1236 cells and U-HO1 cells, and shRNA knockdown of MAOA reduced the growth of L1236 cells. Conversely, ectopic overexpression of MAOA increased the growth of MAOA-negative HDLM2 cells. Combined treatment with clorgyline and ABVD (doxorubicin, bleomycin, vinblastine, dacarbazine) was more effective in reducing cell growth than either regimen alone. These results indicate that targeting MAOA may be an effective approach in cHL.

Another mechanism was studied by Lollies et al. [3], based on the notion that the activator protein-1 (AP-1) factor basic leucine zipper transcription factor, ATF-like 3 (BATF3), is highly transcribed in cHL and anaplastic large cell lymphoma (ALCL). They show that prototypical CD30^+^ lymphomas, namely cHL (21/30) and mDLBCL (8/9), but also CD30^+^ DLBCL (15/20) frequently express BATF3 protein. Mass spectrometry and co-immunoprecipitation established interactions of BATF3 with JUN and JUNB in cHL and ALCL lines. BATF3 knockdown using short hairpin RNAs was toxic for cHL and ALCL lines, reducing their proliferation and survival. Next, they identified MYC as a critical BATF3 target and confirmed binding of BATF3 to the MYC promoter. JAK/STAT signaling regulated BATF3 expression, as chemical JAK2 inhibition reduced and interleukin-13 stimulation induced BATF3 expression in cHL lines. Chromatin immunoprecipitation substantiated a direct regulation of BATF3 by STAT proteins in cHL and ALCL lines. They conclude that in cHL and ALCL STAT-mediated BATF3 expression is essential for lymphoma cell survival and promotes MYC activity, indicating a new oncogenic axis in these lymphomas.

### B cell lymphomas

Batmanov et al. [4] performed whole-genome sequencing in follicular lymphoma (FL) and found three novel highly recurrent regulatory mutation blocks near BCL6 and BCL2, genes known to be important in FL development. They also found the transcription factors whose binding may be disturbed by these mutations: disruption of the FOX-family near the BCL6 promoter results in reduced BCL6 expression. Because of this, there is increased BCL2 expression even to higher levels than when caused by BCL2 gene translocation. Oricchio et al. [5] in a different approach identified *SESTRIN1* as a relevant target of the 6q deletion and demonstrate tumor suppression by *SESTRIN1* in vivo. Moreover, they show that *SESTRIN1* is a direct target of the lymphoma-specific *EZH2* gain-of-function mutation (*EZH2* ^*Y641X*^). *SESTRIN1* inactivation disrupts p53-mediated control of mammalian target of rapamycin complex 1 (mTORC1) and enables mRNA translation under genotoxic stress. *SESTRIN1* loss represents an alternative to *RRAGC* mutations that maintain mTORC1 activity under nutrient starvation. The anti-tumor efficacy of pharmacological EZH2 inhibition depends on SESTRIN1, indicating that mTORC1 control is a critical function of EZH2 in lymphoma. Conversely, *EZH2* ^*Y641X*^ mutant lymphomas show increased sensitivity to RapaLink-1, a bifunctional mTOR inhibitor. They conclude that SESTRIN1 contributes to the genetic and epigenetic control of mTORC1 in FL and might influence responses to targeted therapies.

Although probably all of the literature reviews in this series (at least all in the last year had; [6–9]) are having new information on the role of EBV in malignant lymphoma, this one has more than average. Cohen et al. [10] builds on recent data that EBV-mediated B cell transformation is not only achieved through the action of latent proteins, but that also lytic EBV replication has a certain pathogenic role in lymphomagenesis. They show by real-time quantitative PCR in a series of EBV+ DLBCL from Argentina an unexpected number of cells that express the lytic transcripts BZLF1, BHRF1, and BLLF1. This lytic antigen expression was confirmed by immunohistochemical staining for early lytic protein BMRF1. It is unclear why this does not result in tumor cell killing. Sunagawa et al. [11] investigate EBV-encoded microRNAs (miRNAs) in two regions of its DNA genome, BART and BHRF. They were able to amplify nine DNA fragments encoding miR-BARTs and two coding miR-BHRF1s in 16 cases of EBV-associated lymphoma. An adenine-to-guanine mutation in the DNA fragment encoding miR-BART2-3p was detected in four of the 16 cases, and a cytosine-to-thymidine mutation in the DNA fragment encoding miR-BART11-3p was detected in one of the 16 samples. These mutations were not associated with any histological category of lymphoma. In conclusion, mutations were rarely observed in the DNA encoding viral miRNAs in cases of lymphoma. This suggests that the DNA sequences of EBV-encoded miR-BARTs and miR-BHRF1-1 are conserved in EBV-associated lymphoma. However, controls from reactive processes were not studied. McHugh et al. [12] are interested in dual role of EBV and Kaposi sarcoma-associated herpesvirus (KSHV), which both establish persistent infections in B cells. KSHV is linked to primary effusion lymphoma (PEL), and 90% of PELs also contain EBV. They developed mice reconstituted with human immune system components as a model for KSHV infection and show that EBV/KSHV dual infection enhanced KSHV persistence and tumorigenesis. Dual-infected cells displayed a plasma cell-like gene expression pattern similar to PELs. KSHV persisted in EBV-transformed B cells and was associated with lytic EBV gene expression, resulting in increased tumor formation. Evidence of elevated lytic EBV replication was also found in EBV/KSHV dually infected lymphoproliferative disorders in humans. These data suggest that KSHV augments EBV-associated tumorigenesis via stimulation of lytic EBV replication. Why this results in the peculiar PEL and not other lymphomas remains unclear. Wu et al. [13] investigate EBV-positive DLBCL of the elderly (although it has now been shown that age is not a relevant factor in EBV-positive DLBCL), which accounts for 8–10% of DLBCLs in Asian countries, but is less common in Western populations. Five DLBCL-derived cell lines were used to characterize patterns of EBV latent gene expression, as well as response to cytokines and chemotaxis. Interleukin-4 and interleukin-21 modified LMP1, EBNA1, and EBNA2 expression depending on cell phenotype and type of EBV latent program (type I, II, or III). These cytokines also affected CXCR4- or CCR7-mediated chemotaxis in two of the cell lines. They used dominant-negative EBV nuclear antigen 1 to eliminate EBV genomes, which resulted in decreased chemotaxis. An alternative way to eliminate EBV genomes, Roscovitine, resulted in an increase of apoptosis in the EBV-positive lines. These results show that EBV plays an important role in EBV-positive DLBCL lines with regard to survival and chemotactic response. They conclude that their results provide evidence for the impact of microenvironment on EBV-carrying DLBCL cells and might have therapeutic implications. Yin et al. [14] are also able to promote cell death in EBV-positive cells in vitro, by using arsenic trioxide (ATO). EBV spontaneous reactivation starts as early as 6 h after re-suspending EBV-positive Mutu cells in RPMI media in the absence of ATO; however, this does not occur in Mutu cells cultured with ATO. ATO’s inhibition of EBV spontaneous reactivation is dose dependent. The expression of the EBV immediate early gene Zta and early gene BMRF1 is blocked with low concentrations of ATO in EBV latency type I cells. The combination of ATO and ganciclovir further diminishes EBV gene expression. ATO-mediated reduction of EBV gene expression can be rescued by co-treatment with the proteasome inhibitor MG132, indicating that ATO promotes ubiquitin conjugation and proteasomal degradation of EBV genes. Co-immunoprecipitation assays with antibodies against Zta pull down more ubiquitin in ATO-treated cell lysates. Furthermore, MG132 reverses the inhibitory effect of ATO on anti-IgM-, PMA- and TGF-β-mediated EBV reactivation. Thus, mechanistically ATO’s inhibition of EBV gene expression occurs via the ubiquitin pathway. Moreover, ATO treatment results in increased cell death in EBV-positive cells compared to EBV-negative cells, as demonstrated by both MTT and trypan blue assays. ATO-induced cell death in EBV-positive cells is dose dependent. ATO and ganciclovir in combination further enhances cell death specifically in EBV-positive cells. It would of course be very interesting to see whether these approaches to target EBV in EBV-positive lymphomas are also effective in patients.

Wang et al. [15] combined patient tissue and data with in vitro work to provide a rationale for AKT targeting in DLBCL. AKT signaling is important for proliferation and survival of tumor cells. They assessed expression of phosphorylated AKT (p-AKT) in 522 DLBCL patients and found that high levels (24%) were associated with worse progression-free survival and Myc and Bcl-2 overexpression. However, multivariate analysis indicated that high AKT expression was not an independent prognostic factor. Furthermore, 63 miRNAs directly or indirectly related to the phosphatidylinositol 3-kinase/AKT/mechanistic target of rapamycin pathway were differentially expressed between DLBCLs with high and low p-AKT nuclear expression. The highly selective AKT inhibitor MK-2206 inhibited lymphoma cell viability in 26 DLBCL cell lines. MK-2206 sensitivity correlated with AKT activation status in DLBCL cells. This study demonstrates the clinical and therapeutic implications of high AKT expression in DLBCL and suggests that AKT inhibitors could be combined with other targeted agents for DLBCL.

## Defining entities

### B cell lymphomas

The border between benign and malignant lymphoproliferations is not sharp not really a boundary. Nevertheless, it is important to separate lesions into the two. Méhes et al. [16] tried to distinguish autonomous form regulated proliferation as a new indicator for malignant versus benign lymphoproliferations. Cell kinetic activity is obvious at many levels including progressive chromatin modification and elevated mitotic rates. They used phospho-H3 histone (pH3S10)-specific immunohistochemistry on cell cultures, reactive and selected indolent and aggressive lymphoma samples. They quantified the “dot-type” (representing late G2 phase) and “mitotic” staining and found an accumulation of G2 phase pH3S10 pattern in the reactive germinal centers in contrast to lymphomas. The dot-type G2 staining pattern was also higher in the reactive germinal centers compared to aggressive lymphomas. The relative G2/M value was also higher in reactive germinal center B cells than in any evaluated lymphoma entity, including Burkitt lymphoma. It would be interesting to test this finding in situations where the distinction between chronic inflammation and lymphoma is difficult. Fragkioudaki et al. [17] studied such a situation but with a different approach, namely looking for genetic susceptibility. Like many autoimmune diseases, Sjögren’s disease (SD) is associated with increased lymphoma development at the site of the target organ of the disease. It is therefore quite understandable that the distinction between benign and malignant lymphoproliferations can be difficult. To identify patients who are at very high risk for lymphoma development, they studied two common polymorphisms, the c. 677C > T and c. 1298A > C of the methylene-tetrahydrofolate reductase (MTHFR) gene. This gene encodes for an enzyme essential in DNA synthesis and methylation, and the polymorphisms have been associated with susceptibility to NHL. They genotyped 356 SD patients, of whom 75 had extranodal marginal zone lymphoma (ENMZL) and 19 other types of NHL and 600 healthy controls and found increased frequency of c. 677C > T TT genotype and T allele, as well as reduced prevalence of the c. 1298A > C C allele in the SD other NHL group compared to controls and SD patients without NHL. MTHFR c. 677C > T TT genotype was also associated with reduced DNA methylation levels, while MTHFR c. 1298A > C AC genotype with reduced DNA double-strand breaks levels. Although these findings are interesting, they are not of help in diagnostic hematopathology. Moody et al. [18] used the most classic approach, investigating genetic alterations that may be specific for malignant disease. Both antigenic drive and genetic change play critical roles in the development of ENMZL, but neither alone is sufficient for malignant transformation, and lymphoma development critically depends on their cooperation. They investigated therefore somatic mutations of 17 genes ánd immunoglobulin heavy chain variable region (IGHV) usage in 179 ENMZL lymphomas from various sites. They showed that (1) there was a significant association between the biased usage of IGHV4-34 (binds to the carbohydrate I/i antigens) and inactivating mutation of TNFAIP3 [encoding a global negative regulator of the canonical nuclear factor-κB (NF-κB) pathway] in ocular adnexal ENMZL; (2) IGHV1-69 was significantly overrepresented (54%) in ENMZL of the salivary gland, but was not associated with a mutation in any of the 17 genes investigated; and (3) ENMZL lacked mutations that are frequently seen in other B cell lymphomas characterized by constitutive NF-κB activities, including mutations in CD79B, CARD11, MYD88, TNFRSF11A, and TRAF3. This also separates ENMZL from nodal marginal zone lymphoma (NMZL), as was recently shown by van den Brand et al. [19–21]. The finding that there is a significant association between biased usage of autoreactive IGHV and somatic mutation of NF-κB regulators in ENMZL argues for the hypothesis that the cooperation between antigen receptor binding and genetic changes results in sustained chronic B cell receptor signaling driving oncogenesis in ENMZL development.

Barth et al. [22] investigated the next step in ENMZL development, transformation into DLBCL. They performed cluster analyses on a transcriptome signature of NF-κB target genes of 30 gastrointestinal (GI) B cell NHL (8 ENMZL, 22 DLBCL—among them 9 with coexisting small cell component) and 6 nodal DLBCL (3 activated B cell like (ABC), 3 germinal center-like (GCB)). The GI DLBCL had a distinct pattern which was further confirmed by a cohort of 270 available B cell lymphoma and B cell in silico profiles. Of the NF-κB genes analyzed, c-REL was overexpressed in GI ENMZL. c-REL amplification was limited to 6/22 GI DLBCL including the large cell component of 2/9 composite small cell/large cell lymphomas, and c-Rel protein expression was found in the large cell compartment of composite lymphomas. These data support the concept that GI DLBCL is distinct type from nDLBCL and may arise from ENMZL.

Actually, transformation from low-grade B cell lymphoma to DLBCL is quite common but has distinct features in the different types of low-grade lymphoma. Aukema et al. [23] investigated the role of MYC in the transformation of follicular lymphoma (FL). This lymphoma has an annual risk of transformation of approximately 3% which is associated with aberrations in CDKN2A/B, TP53, and MYC. Like in primary DLBCL, high MYC expression in transformed FL (tFL) might predict a MYC translocation. They quantified MYC expression by immunohistochemistry and digital analysis in 41 paired biopsies from 20 patients with low-grade FL with subsequent transformation and in 4 isolated biopsies of tFL. As controls, 28 biopsies of low-grade FL without transformation (median follow-up 105 months) and 9 grade 3 FL were analyzed. In the 20 pairs, MYC expression was significantly higher in tFL than in the initial FL1/2 biopsies (median 54 vs 6%; 7% in FL3A, 35% in FL3B). MYC breaks (MYC-R+) were detected in 8/21 (38%) by fluorescence in-situ hybridization (FISH). In 2 of the analyzed tFL cases, the translocation was already detected in antecedent FL1/2. MYC partners were immunoglobulin (IG) loci in 3/8 cases (1× IGL, 1× IGH, 1× IGK) and non-IG in 5/8 cases (2× PAX5, 1× BCL6, 2× unknown). Of the 8 MYC-R+ cases, 6 were BCL2+/MYC+ double-hit, 1 a BCL2+/BCL6+/MYC+ triple-hit, and 1 MYC+ single-hit. All 3 IG-MYC-positive cases showed MYC expression > 85%, while the 5 cases with a non-IG MYC partner had a wider range of expression (median 68%, range 13–86%). Three of the 13 MYC-R-negative tFL showed ≥ 90% MYC expression, suggesting alternative mechanisms of MYC activation. MYC is extensively studied, because its involvement in many biological processes. Xiong et al. [24] show that in DLBCL serum metabolomic analysis indicates that oncogenic MYC induces aberrant choline metabolism by transcriptionally activating the key enzyme phosphate cytidylyltransferase 1 choline-α (PCYT1A). In B cell lymphoma cells, as a consequence of PCYT1A upregulation, MYC impeded mitophagy-dependent necroptosis. In DLBCL patient samples, overexpression of PCYT1A was in parallel with an increase in MYC expression, as well as a decrease in serum choline metabolite phosphatidylcholine levels. Both in vitro and in vivo, the lipid-lowering alkaloid berberine (BBR) exhibited an anti-lymphoma activity through inhibiting MYC-driven downstream PCYT1A expression and inducing mitophagy-dependent necroptosis. Maifrede et al. [25] look into another effect, in Burkitt lymphoma. They demonstrate that untreated and cytarabine (AraC)-treated IGH/MYC-positive Burkitt lymphoma cells accumulate a high number of potentially lethal DNA double-strand breaks (DSB) and display low levels of the BRCA2 tumor suppressor protein, which is a key element of homologous recombination (HR)-mediated DSB repair. BRCA2 deficiency in IGH/MYC-positive cells was associated with diminished HR activity and hypersensitivity to PARP1 inhibitors (olaparib, talazoparib) used alone or in combination with cytarabine. Moreover, talazoparib exerted a therapeutic effect in NGS mice bearing primary Burkitt lymphoma xenografts. This very interesting study suggests that IGH/MYC-induced BRCA2 deficiency may predispose Burkitt lymhpoma cells to synthetic lethality triggered by PARP1 inhibitors, which might of course also be relevant for other lymphoma types with similar activation of MYC.

### T cell lymphomas

Anaplastic lymphoma kinase (ALK)-positive anaplastic large cell lymphoma is characterized by 2p23/*ALK* aberrations, including the classic t(2;5)(p23;q35/*NPM1-ALK*) rearrangement present in ~ 80% of cases and several variant t(2p23/ALK) occurring in the remaining cases. The *ALK* fusion partners play a key role in the constitutive activation of the chimeric protein and its subcellular localization. Using various molecular technologies, van der Krogt et al. [26] have characterized ALK fusions in eight recently diagnosed ALCL cases with cytoplasmic-only ALK expression. The identified partner genes included *EEF1G* (one case), *RNF213/ALO17* (one case), *ATIC* (four cases), and *TPM3* (two cases). Notably, all cases showed copy number gain of the rearranged *ALK* gene, which is never observed in *NPM1-ALK*-positive lymphomas. They hypothesized that this could be due to lower expression levels and/or lower oncogenic potential of the variant *ALK* fusions. Indeed, all partner genes, except *EEF1G*, showed lower expression in normal and malignant T cells, in comparison with *NPM1.* In addition, they found that Npm1-Alk has a stronger transformation potential than Atic-Alk, and also observed a subclonal gain of *Atic-Alk* after a longer culture period, which was not observed for *Npm1-Alk.* These data illustrate that lymphomas driven by the variant ATIC-ALK fusion (and likely by RNF213-ALK and TPM3-ALK), but not the classic NPM1-ALK, require an increased dosage of the *ALK* hybrid gene to compensate for the relatively low and insufficient expression and signaling properties of the chimeric gene.

## New entities/subtypes

Perier et al. [27] describe a new lesion, reporting the clinical, virological, and pathology data of five HIV-negative male patients who developed human herpesvirus 8 (HHV-8)-induced tumors following rituximab therapy. HHV-8, also known as Kaposi’s sarcoma (KS)-associated herpesvirus, is involved in KS and other tumors comprising multicentric Castleman disease (MCD) and PEL. Rituximab is currently used for treatment of several autoimmune or inflammatory diseases and humoral organ rejection. Their five patients were all immunocompromised by previous treatments, with steroids and/or immunosuppressive therapy requiring rituximab for disease progression or rejection. The patients developed HHV-8 tumors in a median time of 6 months (range 3 to 13) after rituximab. Four patients had at least one risk factor of HHV-8 including high Fitzpatrick skin phototype > 3 (*n* = 3) and homosexuality (*n* = 1). Four patients developed KS with skin lesions (*n* = 4) and visceral involvement (*n* = 2) and one patient developed a solid PEL. Rituximab was withdrawn in all patients and immunosuppression was reduced when feasible. After a median follow-up of 20 months, two patients died. Remission of KS was complete (*n* = 1), partial (*n* = 1) and one patient had progression. They conclude that safety of rituximab especially in non-lymphomatous disorders has to be carefully evaluated in patients at risk for HHV-8 positive tumors.

The prognosis of the CD8^+^ subtype of mycosis fungoides (MF) is controversial. Although most authors believe that determining the presence of this cell surface antigen has no prognostic value, others have observed a more indolent course for CD8^+^ MF compared with CD4^+^ MF. Martinez-Escala et al. [28] collected 67 patients (median age 46 years) with CD8+ MF and found higher frequency of early-stage disease compared with other cohorts, including 31 (47%) patients with stage IA, 33 (50%) with stage IB, and 2 (3%) with stage IIB. With a median follow-up (5.5 years) similar to other cohorts, a higher rate of complete remission was achieved (66%), and a lower rate of progression was observed. These results indicate a more indolent course of disease for CD8+ MF which warrants a conservative treatment approach limited to skin-directed therapies and observation in most patients.

## Pitfalls in lymphoma diagnosis

Haacke et al. [29] indicate that the recognition of ENMZL on labial gland biopsies can be difficult especially in patients with primary Sjögren’s syndrome (pSS), who have an increased risk of developing ENMZL. The presence of germinal centers (GCs) in labial gland biopsies has been suggested as predictive factor for the presence of NHL. Eleven labial gland biopsies from patients with pSS that were taken prior to parotid ENMZL development were compared with biopsies of 22 matched pSS controls who did not develop lymphoma. Labial gland biopsies of pSS ENMZL patients revealed GCs in 2/11 (18%) H&E sections and in 3/11 (27%) Bcl6-stained sections. In the controls, GCs were present in 4/22 (18%) of H&E sections and 5/22 (23%) of Bcl6-stained sections. These results indicate that presence of GCs in labial gland biopsies does not differentiate between patients with pSS who develop parotid ENMZL and patients with pSS who do not.

Patterns of expression of markers are very helpful in classifying B cell lymphoma, but can be deceptive too, because of 2 reasons. In the first place, cancer cells can have abnormal expressions and cells, including cancer cells, can change their expression pattern when they come into a different environment. Pizzi et al. [30] aimed to define the epidemiological, histological and cytogenetic characteristics of BCL6 and CD10-positive mantle cell lymphoma (MCL). They found in 165 cases of cyclin D1 and t(11;14)(q13;q34)-positive MCLs BCL6 and CD10 expression in 26 of 165 (16%) cases (BCL6 17 of 165; CD10 11 of 165; BCL6 and CD10 co-expression 2 of 165). Either expression correlated significantly with a higher proliferation index and higher prevalence of MUM1 positivity. Fluorescence in-situ hybridization (FISH) for the BCL6 (3q27) gene showed amplifications more frequently in BCL6-positive than BCL6-negative cases (50.0 versus 19% of cases). The authors conclude that aberrant CD10 and BCL6 expression defines a subset of MCLs with higher mean Ki-67 index and higher prevalence of MUM1 expression. However, this is not tested as an independent factor and might actually be due to abnormal expression in the environment, like in NMZL as is recently described by van den Brand et al. [31].

Cutaneous T Cell Lymphomas (CTCL) are rare, but potentially devastating malignancies, whose pathogenesis remains poorly elucidated. Unfortunately, currently, it is not possible to predict in which patients the process will progress and which patients will experience an indolent disease course. Furthermore, at early stages, this malignancy often masquerades as psoriasis, chronic eczema, or other benign inflammatory dermatoses. As a result, it takes on average 6 years to diagnose this lymphoma after the initial presentation. Litvinov et al. [32] performed transcription expression profiling using TruSeq-targeted RNA gene expression on 181 fresh and formalin-fixed and paraffin-embedded (FFPE) skin samples from cutaneous T cell lymphoma (CTCL) patients and patients affected by benign inflammatory dermatoses that often mimic CTCL clinically and histologically (e.g., psoriasis, chronic eczema, etc.) Their results underscore significant molecular heterogeneity with respect to gene expression between different patients and even within the same patient over time. The study also confirms that *TOX*, *FYB*, *LEF1*, *CCR4*, *ITK*, *EED*, *POU2AF*, *IL26*, *STAT5*, *BLK*, *GTSF1*, and *PSORS1C2* are genes being differentially expressed between CTCL and benign skin lesions. In addition, they found that differential expression for a subset of these markers (e.g., *TOX*, *FYB*, *GTSF1*, and *CCR4*) may be useful as prognostic indicator for this disease. Although promising, it is not an easy approach, which makes it difficult to use in routine practice. Pereira et al. [33] collected 15 cases of pseudolymphomatous cutaneous lupus erythematosus (cLE) other than lupus panniculitis and lupus tumidus (M to F = 4 to 11, age range 23–79 years, mean age 51 years). Of the 15 cases, 9 (60%) were characterized by dense nodular infiltrates. Three cases (20%) showed an angiocentric pattern with cytological atypia of lymphoid cells; 2 cases (13%) showed a band-like infiltrate mimicking MF, and 1 case had mixed features of the band-like and angiocentric patterns. Clues to the histopathological diagnosis of cLE were presence of interface dermatitis, clusters of plasmacytoid dendritic cells, and dermal mucin deposition. This study shows that the spectrum of pseudolymphomatous presentations of cLE is broader than previously described, including band-like cases that may be misdiagnosed as MF, and angiocentric cases that may be misinterpreted as an aggressive lymhpoma. Recognition of such cases is possible only on careful clinicopathologic correlation and requires a high level of histopathological suspicion to allow a correct diagnosis and the proper management of the patients. However, on the same token, Nguyen et al. [34] try to give a clue towards a pathological distinction between benign and malignant lymphoid infiltrates in the skin. They analyzed skin biopsies from 42 prelymphomatous T cell dyscrasias (CLD), 9 Sezary’s syndrome (SS), 103 MF, and 20 CD30+ lymphoproliferative diseases (LPD) for programmed death marker-1 (PD-1) expression using immunohistochemistry. PD-1 staining was observed amidst many neoplastic T cells in 6/9 (67%) and 62/103 (60%) cases of SS and MF, respectively, while only 6/42 (14%) cases of CLD and 0/20 (0%) cases of CD30+ LPD. Three cases are from same patients representing different stages of disease evolution from CLD to MF and SS with a corresponding enrichment of PD-1 positivity. In all cases, there was variable staining of PD-1 amidst macrophages. There was no correlation with disease progression among MF cases. Therefore, PD-1 correlates with disease progression in epitheliotropic T cell proliferations, ranging from minimal staining in prelymphomatous lesions to significant staining in MF. Because PD-1 was consistently expressed in MF while it was consistently negative in primary CD30+ LPD, this indicates the possibility of using PD-1 as a means of distinguishing CD30+ MF from primary cutaneous ALCL. If and when confirmed, these findings may actually be of real help in our practice.

## Prognostic factors in lymphoma

As usual, a small outtake of the articles providing prognostic information on lymphoma types. Currently, we see more emphasis on the tumor microenvironment than previously. Koh et al. [35] used diagnostic tissues from 135 cHL patients treated with ABVD and found that the presence of EBV and VEGF expression were positively correlated. EBV-positive patients had a lower 5-year overall survival rate than EBV-negative patients. A high microvascular density was also associated with a poorer overall survival, which held up in multivariate analysis, in contrast to EBV-positivity. Meirav et al. [36] found that in 48 patients with FL, high extrafollicular PD1 expression predicted superior freedom from disease progression compared to low expression (at 5 years 52 vs 44%); low intrafollicular CD3 expression also indicated better outcome (at 5 years 37 vs 67%). Gong et al. [37] collected a total of 127 patients with aggressive B cell lymphoma and looked for prognostic indicators among clinical features, as well as CD4, Foxp3, CD8, CD68, CD163, PD-1, and PD-L1 expression as assessed using immunohistochemistry. Significant differences in 11 factors (age, Ann Arbor stage, B symptom, ECOG performance status, infiltrating CD8+ T cells, PD-L1 expression, absolute blood monocyte count, serum lactate dehydrogenase, serum iron, serum albumin, and serum β2-microglobulin) were observed among patient groups stratified by at least two risk stratification methods [International Prognostic Index (IPI), revised IPI, and NCCN-IPI models]. Concordance rates were high (81.4–100.0%) when these factors were used for the risk stratification. Although the authors conclude that this results in an easy and inexpensive tool, it seems to me not at all easy, and therefore I am doubtful that this will enter the clinic. As described above, MYC is relevant in several lymphomas, but little data indicate its relevance in mantle cell lymphoma (MCL). Nevertheless, Gong et al. [38] found in 64 patients with MCL differences in cytoplasmic C-MYC expression, Ki-67 proliferative index of tumor cells, and CD8 positive tumor-infiltrating lymphocytes (CD8 + TIL) among three risk groups; no significant differences existed in the expression of nuclear C-MYC, SOX11, p53, and PD-L1. As expected, patient age and serum LDH level were also significantly different among the 3 groups of patients. It remains unclear how this could be helpful in treatment decisions.

DLBCL has been studied the most for prognostic markers. Quesada et al. [39] look into MYC, which has been studied in many different ways already. They studied 663 patients with de novo DLBCL in whom the status of MYC/8q24, BCL2/18q21, and BCL6/3q27 was assessed by FISH. In 76 patients, they found extra copies of MYC, including 43 cases of double or triple extra copies; 105 patients had MYC-rearrangement (R) including 56 double- or triple-hit lymphoma, and 482 had no MYC abnormality (MYC normal). Patients with MYC extra copies, similar to MYC-R, had a worse overall survival compared with MYC normal patients. The prognosis between patients with MYC extra copies and MYC-R was not different. Cell-of-origin classification failed to correlate with survival in the MYC extra copies group, similar to the MYC-R patient group. Multivariate analysis showed that MYC extra copies in DLBCL are an independent poor prognostic factor, similar to MYC rearrangement. Xie et al. [40] investigate the role of ICT1, immature colon carcinoma transcript-1, a crucial member of the large mitoribosomal subunit in mitochondrial ribosome, which has been shown to be closely related to tumorigenesis. This study started with an analysis of the public available Oncomine database in which they found that the expression level of ICT1 mRNA was significantly upregulated in DLBCL tissues. Next, they confirmed that ICT1 was upregulated in fresh DLBCL samples compared with the corresponding normal tissues using quantitative reverse-transcription polymerase chain reaction and Western blotting. ICT1 overexpression was associated with poor overall survival. Finally, they found in DLBCL cell lines that shRNA-mediated knockdown of ICT1 suppressed proliferation, induced cell cycle arrest at G0/G1 phase, and apoptosis. Further verification showed that inhibition of ICT1 gene expression caused the upregulation of the p21, Bad and caspase-3, and downregulation of PCNA, Survivin, CDK4, CDK6, and Cyclin D1. Taken together, this study suggests that ICT1 may play an oncogenic role in DLBCL by promoting cell proliferation and it might be a biomarker for unfavorable prognosis in DLBCL patients.

Xu et al. [41] show that junctional adhesion molecule-A (JAM-A) was highly expressed in DLBCL patients with multiple extranodal lesions. JAM-A maintains B lymphoma cell stemness and was associated with cell invasion and epithelial-to-mesenchymal transition both in vitro and in vivo. As mechanism of action, JAM-A overexpression selectively activated transforming growth factor-β (TGF-β)/NODAL signaling, thereby enhancing B lymphoma cell aggressiveness and inducing extranodal involvement to mesoendoderm-derived organs in DLBCL. Lenalidomide downregulated JAM-A and downstream NODAL expression, resulting in inhibition of B lymphoma cell invasion and epithelial-to-mesenchymal transition. In a murine xenograft model established with subcutaneous injection of JAM-A-overexpressing B lymphoma cells, lenalidomide retarded tumor growth and prevented cell invasion to mesoendoderm-derived organs, consistent with the downregulation of JAM-A and NODAL expression. Collectively, these findings indicated that JAM-A is related to en involvement in DLBCL through modulating TGF-β/NODAL signaling. Therapeutic targeting of JAM-A/NODAL axis could thus be a promising clinical strategy for DLBCL patients.

## Ancillary techniques

Distinction between GCB- and ABC-type DLBCL remains difficult in routine practice, since the several algorithms based immunohistochemistry are not reliable enough. Phang et al. [42] used one of the recent developed RNA-based platforms (Lymph2Cx) on diagnostic biopsies. Of the 104 samples, 96 (92%) were informative and of these, 38 were labeled as GCB (40%) and 58 were non-GCB (60%) by Hans algorithm. Lymph2Cx cataloged 36/96 (37%) samples as GCB, 45/96 (47%) as ABC, and 15/96 (16%) as unclassified. Using Lymph2Cx as reference, IHC protocol revealed sensitivity of 81% for ABC and 75% for GCB and positive predictive value of 81 versus 82%, respectively. Lymph2Cx-based COO classification performed superior to the Hans algorithm in predicting survival.

Fontanilles et al. [43] investigated whether cell free (cf) DNA obtained from blood samples might be a reliable source for testing for somatic mutations in primary central nervous lymphoma (PCNSL). They compared results of targeted sequencing of plasma cfDNA and matched tumor DNA (tDNA) from 25 PCNSL patients. According to the patient-specific targeted sequencing, eight patients (32%) had detectable somatic mutations in cfDNA. Considering MYD88 sequencing, six patients had the specific c.T778C alteration detected in plasma. Using a control group of 25 age-matched healthy controls, the sensitivity was 24% and the specificity was 100%. Tumor volume or deep brain structure involvement did not influence the detection of somatic mutations in plasma. This pilot study provides evidence that somatic mutations can be detected by NGS in the cfDNA of a subset of patients with PCNSL with high specificity.
